# Some Diseases of the Female Urethra

**Published:** 1913-10

**Authors:** W. F. Shallenberger

**Affiliations:** Atlanta, Ga.


### SOME DISEASES OF THE FEMALE URETHRA.

By Dr. W. F. Shallenberger, Atlanta, Ga.

The female urethra is frequently the seat of a pathological condition that may be unrecognized or neglected. It is for the purpose of calling your attention to some of the facts in this connection that I have chosen this subject for my paper.

Before we take up the pathological discussion I shall give a brief review of some of the anatomical points. The female urethcra is short and corresponds to that portion of the male urethra which extends from the bladder to the ejaculatory ducts. It runs for a distance of 3½ to 4 cm. downward and forward from behind the symphysis and arch of the pubis to the vestibule where the external meatus lies below the clitoris, between the labia minora and above the vaginal orifice. The external meatus is a vertical slit in younger women which is formed by the apposition of the urethral labia, two distinct lips which come together and fit into the meatus, closing it like a valve. In older women and women who have borne children these labia are usually absent. This office is to protect the urethra from infection without.

The submucosa is rich in fibroelastic tissue interwoven with muscle fibres from the muscular layers, which lie outside the submucosa. There are two planes of smooth and two planes of striated muscle fibres. The vesical sphincter lies as a ring of smooth muscle fibres about the internal orifice. The urethra is capable of considerable distension.

In the tissue about the urethra is a plexus of large veins analogous to the corpus spongiosum of the male.

The mucous membrane of the urethra is composed of stratified squamous epithelium which becomes somewhat transitional toward the internal orifice. It lies in longitudinal folds when the urethra is at rest. There are a numebr of small glands which open in the creases between the folds along the posterior wall or floor of the urethra. These glands are mostly small, tubular cysts but some become racemose, especially toward the external meatus and resemble the glands of Littre in the male urethra.

At or near the external meatus on the floor of the urethra, are found the openings of so-called Skenes glands, one on either side. They are the paraurethral glands, and lie beneath the urethra and parallel to it for ½ inch. They harbor infection and may give rise to sub-urethral abscesses and cysts.

The mucosa has a pale pink color normally and as it closes over the end of an endoscope it has a striated appearance, longitudinally.

The diseases of the female urethra are those pathological conditions which are peculiar to mucous membranes, inflammations, new growths, etc. I shall not consider anomalies. Insignificant as the urethra may seem in size and simplicity of structure it may be the cause of intense suffering and a very simple condition may give rise to years of suffering, frequently because the physician has not recognized the trouble. The symptoms are not always typical and may not be referred to the urethra. Where a patient complains persistently and consistently of pain in the region of the pelvis and no other pathological condition can be found to account for it, the urethra should always be thoroughly investigated.

New Growths.—Primary carcinoma is rare though it may occur at any point of the urethra. Secondary invasion from without is more common. Unless seen early it may be impossible to determine the origin of a carcinoma which involves the urethra.

Sarcoma in very rare and only about four cases have been reported in the literature.

The most frequent new growth is the carulcle. This is a highly vascular tumor of small size, as a rule, springing from the wall of the urethra near the meatus, and often projecting from the meatus. It is bright red in appearance and almost always exquisitely tender and sensitive to the slightest touch. The pain on urination may be excessive.

At least three different varieties of caruncle have been described. The granuloma, which is the sequel to a gonnorrheal infection: the papillary angioma and the non-papillated telangiectatic type. They may be pedunculated or sessile, and occur usually in middle life. Unless they are completely excised they tend to recur, but there is no maligancy about them.

A condition which is frequently mistaken for a caruncle is prolapse of the urethral mucosa. I have noted this condition in several patients who have had a previous gonorrhea. The prolapsed mucosa is never so bright red as the caruncle. Usually it is a deep red or bluish color and the expuisite tenderness of the caruncle is not present. A careful examination will soon show the broad base of the projecting membrane and differentiation is easy.

Strictures of the female urethra are not very frequent. They may follow sub-urethral abscesses in the glands, ulcers, trauma from calculus, childbirth, etc.

Inflammations are the most common affections of the urethra. Irritation from very acid or very alkaline urine may set up an inflammatory process. This is often observed in warm weather when the patient has been perspiring freely and has not taken much water, and the urine becomes concentrated. In some of the acute infectious diseases the urethra may be affected. Scarlet fever, measles, etc.

Acute urethritis usually is of gonorrheal origin though it may occur as a downward extension from a cystitis or extension upward from a vulvitis. New growths, and trauma from calculus or childbirth may bring on an acute inflammation of the urethra.

But the point I wish especially to emphasize is the relative frequency with which chronic urethritis occurs in women. This condition may give rise to a great deal of discomfort and suffering. It is often mistaken for a chronic cystitis and many women have their bladders irrigated and dilated daily without relief when the trouble is in the urethra.

The irritation from the repeated use of the catheter aggravates conditions. A chronic urethritis often follows a cystitis and a chronic cystitis may accompany it. Where such is the case the cystitis is usually treated and the urethritis neglected, whereas the urethritis is most likely accountable for most of the pain and discomfort. The more I see of these chronic conditions in the lower female urinary tract the more I am convinced that we should direct our attention more carefully to the urethra in our treatment.

The etiological factor in many of these cases of urethritis is obscure. Where we have a history of a pre-existing cystitis or acute urethritis, the evidence is clear. But many times the trouble develops without any preceding local symptoms. Several years ago Dr. Hunner pointed out the fact that ureteritis and urethritis were not uncommon in women as sequelae to chronic infections elsewhere, such as infected tonsils, nose and ear infections, etc. I have noted the trouble a number of times in patients suffering from excessive intestinal putrefaction. The urethritis in this class of parents, that is, those with other conditions as the exciting cause, will yield, as a rule to local treatment but it will recur usually unless the original source of the trouble is relieved.

Urethral neurosis was a term formerly in common use as a haven of refuge when we failed to find any better name for the trouble. The majority of the patients who suffer from urethral neurosis or irritable urethra probably had nothing more or less than unrecognized chronic urethritis.

In this condition the urethra is thickened throughout as a rule and can be felt through the vagina as a more or less firm cord. Through the endoscope the mucosa has a congested apperance or it may appear granular, or the vessel may be enlarged and exposed. Ulcers may be found. The congested, granular mucosa is the most frequent type with which we meet.

The most constant symptoms are frequent and painful burning urination. In the more severe cases there may be more or less constant pain of a throbbing character in the region of the urethra or vagina, or neck of the bladder. Some patients will have hard attacks with sharp lancinating pains in the urethra. With these pains there may be a constant desire to urinate and even vesical tenesmus. Reflex pains may be present, referred to the vagina, uterus, coccyx, etc. Dr. Hunner says of chronic urethritis: "The symptoms are most protean, and when a patient complains of persistent symptoms of whatever character, located anywhere between the coccyx and the umbilicus, for which no other anatomic lesion can be found, one must never overlook the urethra."

Treatment in these cases consists of applications of silver nitrate solutions to the urethra through an endoscope. I use solutions of from 4% to 10% and repeat the treatments every three to five days. The use of sounds or dilators in addition to this treatment is often also beneficial and massage along the urethra through the vagina with the sound in place may help. But touching up with the silver nitrate alone will usually give results.

During the last two years I have had thirty-two patients who had chronic urethritis. Eight of these had a simple urethritis without any other accompanying trouble and without the history of a pre-existing cystitis or acute purulent urethritis. One patient had a ureteritis in addition to the urethritis. Sixteen had a pre-existing cystitis or some form of chronic cystitis at the time I saw them. Two had incontinence of urine due to relaxed sphincter. Four of the thirty-two were sent to me for diagnosis alone and I did not treat them. Two more stopped treatment before any results had been obtained. Of the remaining twenty-six that I treated eighteen have been cured. The others have all been relieved and six of them are still under treatment. The number of treatments necessary to effect a cure varies with each case.

Four to eight applications of silver nitrate will sometimes suffice, and again a patient may have to come for a much longer period.

One of these patients, referred to me by Dr. McRae, had suffered from a urinary infection for ten or twelve years. There are at present a colon bacillus cystitis and left pyelitis. She has received treatment at various times during her trouble from at least a half dozen doctors. The bladder had been irrigated, retention catheters had been inserted, urotropin and other urinary antiseptics had been tried and re-tried, with no results and during all this time the urethra had never been touched. The cystitis still exists, the pyelitis still is present, but the patient no longer has any pain. By the simple treatment of the urethra the pain and suffering have been relieved. This patient is very large and objects strenuously to urethral catheterizations. I have tried pelvic lavage a couple of times to relieve the pyelitis but she will not submit to it further. I am now using an autogenous vaccine and hope to get some results. If she omits urethral treatments for any period, the pain begins to return and this will be the case until we can clear up the colon bacillus infection.

One patient who had very severe attacks at times which had necessitated the use of morphia in order to get relief, had the bladder irrigated for several months with no results, was cured by eight treatments. This was a year ago and she has remained well ever since.

In conclusion, I wish to urge that you be more attentive to the female urethra and especially where other treatment is not giving results, do not overlook the fact that the trouble may lie in the urethra.

